# SPME Method Optimized by Box-Behnken Design for Impact Odorants in Reduced Alcohol Wines

**DOI:** 10.3390/foods7080127

**Published:** 2018-08-10

**Authors:** Bithika Saha, Rocco Longo, Peter Torley, Anthony Saliba, Leigh Schmidtke

**Affiliations:** 1 National Wine and Grape Industry Centre, Charles Sturt University, Wagga Wagga, NSW 2650, Australia; bithika04@yahoo.com (B.S.); rocco.longo@utas.edu.au (R.L.); peter.torley@rmit.edu.au (P.T.); asaliba@csu.edu.au (A.S.); 2 School of Agricultural and Wine Sciences, Charles Sturt University, Wagga Wagga, NSW 2650, Australia; 3 School of Psychology, Charles Sturt University, Wagga Wagga, NSW 2650, Australia

**Keywords:** reduced-alcohol wine, solid-phase microextraction, gas chromatography, chemometrics

## Abstract

The important sampling parameters of a headspace solid-phase microextraction-gas chromatography-mass spectrometry (HS-SPME-GC-MS) procedure such as the extraction temperature, extraction time, and sample volume were optimized to quantify 23 important impact odorants in reduced alcohol red and white wines. A three-factor design of Box-Behnken experiments was used to determine the optimized sampling conditions for each analyte, and a global optimized condition at every ethanol concentration of interest determined using a desirability function that accounts for a low signal response for compounds. Shiraz and Chardonnay wines were dealcoholized from 13.7 and 12.2% *v*/*v* ethanol respectively, to 8 and 5% *v*/*v*, using a commercially available membrane-based technology. A sample set of the reduced alcohol wines were also reconstituted to their natural ethanol level to evaluate the effect of the ethanol content reduction on volatile composition. The three-factor Box-Behnken experiment ensured an accurate determination of the headspace concentration of each compound at each ethanol concentration, allowing comparisons between wines at varying ethanol levels to be made. Overall, the results showed that the main effect of extraction temperature was considered the most critical factor when studying the equilibrium of reduced alcohol wine impact odorants. The impact of ethanol reduction upon the concentration of volatile compounds clearly resulted in losses of impact odorants from the wines. The concentration of most analytes decreased with dealcoholization compared to that of the natural samples. Significant differences were also found between the reconstituted volatile composition and 5% *v*/*v* reduced alcohol wines, revealing that the dealcoholization effect is the result of a combination between the type of dealcoholization treatment and reduction in wine ethanol content.

## 1. Introduction

Increasing medical and social behavior issues associated with immoderate alcohol consumption have led the World Health Organization (WHO) to introduce, in 2010, a global strategy to reduce the alcohol intake in the population [1]. This has directed many governments to enforce pricing and taxation policies on alcohol production, with the wine sector being financially impacted [2]. Alcohol is also the main source of caloric content in wine and nutritional mandatory labeling occurs in many countries such as South Africa, France, and Germany [3]. Although warning health labeling in some countries such as the United States is currently voluntary, for large chain restaurants nutritional labeling is likely to be mandatory for all foods and beverages served. This development is likely to impact the export market with analytical information as well as a reason to reduce the alcohol content in wine to be competitive with other products. All these considerations have stimulated a greater interest among researchers in order to implement new technologies for reducing the alcohol content in wine [4,5,6]. Several techniques have been experimented to reduce the wine alcohol content, but dealcoholization (i.e., removal of alcohol from wine) using membrane-based devices remains the most employed technique at a commercial scale. From the literature, dealcoholization is also one of the most studied techniques in the field, in part because ethanol removal causes significant losses of important impact odorants, which researchers are committed to minimizing [7].

Impact odorants such as ethyl esters, acetates of higher alcohols, and terpenes, are of primary importance for understanding the consumer acceptability of wines, due to their contribution to desirable aroma attributes at very small concentrations (10^−4^–10^−12^ g/L) [8,9]. Several extraction techniques such as distillation, solvent extraction, and solid-phase have been used prior to the analysis of these volatile compounds; however, headspace solid-phase extraction (HS-SPME) is currently the most used for dealcoholized wine trials [7]. Sampling headspace vapors by SPME essentially includes two operating steps: (i) partitioning of analytes between the fiber coating and the headspace gas phase, and (ii) desorption of the concentrated analytes into an analytical instrument such as a gas chromatograph-mass spectrometer (GC-MS). Compared to conventional extraction techniques, SPME is relatively simple, fast, and cost-efficient. Moreover, SPME requires no-solvent and a limited manipulation of the sample [10,11].

Despite the advantages of SPME, comparisons of HS-SPME-GC-MS results between two or more treatments (e.g., natural and dealcoholized wines) may be confounded by varying experimental conditions, such as the fiber exposure time, sample temperature and volume, and the type and uniformity of the sample matrix including the ethanol content [12,13,14]. In particular, ethanol, the main component of the alcohol content of wine, has a great impact on the partitioning coefficient of other volatile compounds and, thus, on their concentration in the wine headspace [15]. In addition, the competition between ethanol and other analytes for binding sites on the SPME fiber during headspace sampling will be altered with varying sample ethanol concentrations, which may confound characterization studies and may lead to the misinterpretation of results [16]. Optimization of experimental conditions using multivariate statistical approaches such as full three-level factorial Box-Behnken designs, a response surface methodology, helps to overcome these problems [17]. Nevertheless, Box-Behnken designs have not been widely used for the optimization of SPME methods [18].

The aim of this study was to optimize an SPME-GC-MS method to identify and quantify important volatile compounds in red and white reduced alcohol wines. In addition to evaluating the overall effect on reduced alcohol wine’s volatile composition, a sample set of the reduced-alcohol-wines were reconstituted to their natural ethanol level to evaluate the effect of the ethanol content reduction on the volatile composition. A full three-factor Box-Behnken design was used for the optimization of SPME conditions. The quantitative methodology was validated for 23 impact odorants of relevance in wines at each ethanol concentration of interest, alleviating a requirement for sample dilution prior to analysis. This approach removed the confounding effects of varying the ethanol levels upon the partitioning of target compounds from liquid to headspace during the analysis, enabling a more robust comparison of volatile composition at the treatment level.

## 2. Materials and Methods

### 2.1. Chemicals

Ethyl butyrate, ethyl-2-methyl butyrate, ethyl-3-methyl butyrate, isoamyl acetate, 3-methyl-1-butanol, ethyl hexanoate, ethyl-s-lactate, (z)-3-hexenol, methyl octanoate, ethyl octanoate, propanoic acid, linalool, methyl decanoate, ethyl decanoate, isoamyl octanoate, 3-(methylthio)-1-propanol, β-phenyl ethyl acetate, ethyl dodecanoate, geraniol, β-phenyl ethanol, octanoic acid, decanoic acid, and vanillin were purchased from Fulka (Buchs, Switzerland). The analytes’ identification numbers for the Box-Behnken design, odors and olfactory thresholds, and boiling points of each compound are presented in Table 1. Ethanol (VWR Prolabo, Fontenay Sous Bois, France), l-(+) tartaric acid (Sigma, Steinheim, Germany), and potassium hydrogen tartrate (BHD Chemicals Ltd., Poole, UK) were used for the preparation of the model wine solutions. The deionized water was obtained from a Milli-Q mixed bed resin system (18 MΩ/cm, 25 °C).

The internal standards comprising of 2-octanol, 4-methyl-2-pentanone, and deuterated isotopes of ethyl butyrate, ethyl hexanoate, ethyl octanoate, ethyl decanoate, and phenol were used for the preparation of the calibration curves for the target compounds (Tables S3–S5). The deuterated ethyl esters were prepared in accordance with the published methods [19] and the deuterated phenol was obtained from the Polymer Source (Dorval, QC, Canada). An internal standard mix containing 2-octanol (1.94 μg/mL), 4-methyl-2-pentanone (99 μg/mL), d5-ethyl butyrate (8.08 μg/mL), d5-ethyl hexanoate (9.12 μg/mL), d5-ethyl octanoate (3.78 μg/mL), d5-ethyl decanoate (3.74 μg/mL), and d6-phenol (20.26 μg/mL) was prepared in ethanol and stored at −80 °C prior to use.

### 2.2. Instrumentation

Instrumentation and basic analytical conditions were based upon previously published methods for HS-SPME GC-MS analysis of wine volatile compounds [22]. An Agilent 7890A (Agilent Technologies, Ltd., Palo Alto, CA, USA) gas chromatograph, equipped with a Gerstel multipurpose sampler with automated SPME capability and Peltier cooled sample tray, interfaced to an Agilent 5975C (Agilent Technologies, Ltd.) triple axis mass detector, was used for confirmation of the compound’s identity, method development, and final sample analysis. MSD ChemStation^®^ E.02.00.493 (Agilent Technologies, Ltd.) and NIST MS Search 2.0, version 2008, (Agilent Technologies, Ltd.) were used to control the instrument performance and for mass spectra assessment. Samples (10 mL) were placed into the Peltier cooler tray set at 8 °C until analysis, whereupon the vials were transferred to a heater block with a 2-min pre-incubation time before the insertion of the SPME fiber. A fused silica capillary column (DB-Waxetr, 60 m × 0.25 mm inner diameter, 0.25 μm film thickness, J&W Scientific, Folsom, CA, USA) was used for compound separation by GC. The injector block was fitted with a 2-mm internal diameter borosilicate liner (SGE) and the injector temperature was set to 260 °C in the splitless mode. The fiber was inserted into the injector for 1 min, withdrawn and injected into a second injector set at 270 °C with a 50:1 split for 10 min with a 15 mL/min purge flow to clean the fiber prior to the next sample analysis. The oven temperature program commenced at 40 °C for 5 min and increased to 230 °C at a rate of 6 °C/min. The total run time was 45 min. The flow rate of ultra-high-purity helium gas was constant at 3 mL/min. The MS source, quadrupole, and transfer line temperatures were set to 230, 150, and 275 °C, respectively.

### 2.3. Compound Identification and Elution Profiles

The solutions of 23 compounds were prepared at an approximate midpoint of the calibration range, in a model wine solution containing tartaric acid (0.008 M), potassium hydrogen tartrate (0.011 M), and ethanol (5% *v*/*v*) to confirm the compound elution times and ion profiles. Samples (10 mL) were transferred to the heater block set at 50 °C with an agitation rate of 250 revolutions per minute (rpm) and allowed to equilibrate for 1 min. A 23 gauge 50/30 µm divinylbenzene/carboxen/polydimethylsiloxane (DVB/CAR/PDMS) fiber (Supelco, Bellefonte, PA, USA) was preconditioned at 270 °C for 60 min, before insertion into the GC injector. Mass spectral data was collected in selective ion monitoring (SIM) at an ionization voltage of 70 eV. The final elution profiles were confirmed by matching mass spectral data with the NIST mass spectral search program (version 2.0, National Institute of Standards and Technology, Gaithersburg, MD, USA), and Kovat’s retention indices (RIs). RIs were checked for each compound using a commercial mixture of *n*-alkanes (Sigma, Steinheim, Germany), an identical oven ramp profile, and gas flow rates, as used for the final analyses. This formula was used to calculate RI [23]:

RI = 100*z* + 100 (*t*_R(i)_ − *t*_R(z)_/(*t*_R(*z*+1)_ − *t*_R(*z*)_) (1)

RI = relative index of compound i;
*z* = carbon number of the alkane *z*;
*t*_R(i)_, *t*_R(*z*)_, and *t*_R(*z*+1)_ = retention times of the compound *i*, the compound *z*, and the alkane *z* + 1, respectively.

### 2.4. Optimization of Sample Extraction Conditions

Three model wines were prepared with tartaric acid (0.008 M), potassium hydrogen tartrate (0.011 M), and ethanol (5, 8 or 13% *v*/*v*), with the compounds of interest added at concentrations that approximately match the mid-point of the calibration curves. Sample extraction temperature (°C), time (min), and volume (mL), in addition to the ethanol content (% *v*/*v*), were optimized using a three-factor Box-Behnken design (Table 2). The optimum sample incubation temperature, SPME fiber exposure time, and sample volume combinations at each ethanol concentration were based on a previously described method [13]. A quadratic equation with second-order interactions for the three factors was used to determine the maximum predicted signal for each analyte using the following equation:

ŷ = *b*_0_ + *b*_1_*x*_1_ + *b*_2_*x*_2_ + *b*_3_*x*_3_ + *b*_12_*x*_1_*x*_2_ + *b*_13_*x*_1_*x*_3_ + *b*_23_*x*_2_*x*_3_ + *b*_11_*x*_1_^2^ + *b*_22_*x*_2_^2^ + *b*_33_*x*_3_^2^ (2)

ŷ = predicted response; *b*_0_ is the intercept or average response;
*b*_1_*x*_1_ + *b*_2_*x*_2_ + *b*_3_*x*_3_ = linear terms associated with each factor (temp, time, sample vol.);
*b*_12_*x*_1_*x*_2_ + *b*_13_*x*_1_*x*_3_ + *b*_23_*x*_2_*x*_3_ = second-order interaction terms between each factor;
*b*_11_*x*_1_^2^ + *b*_22_*x*_2_^2^ + *b*_33_*x*_3_^2^ = quadratic terms for each factor;
*x*_1_ = factor extraction temperature;
*x*_2_ = factor extraction time;
*x*_3_ = factor sample volume in a 20 mL vial.

The significance of each experimental factor coefficient (*b*) in the quadratic equations was determined using a Students *t*-test following the calculation of a coded design matrix and the predicted responses and residuals [24], as described in the Supplementary Materials (Tables S1 and S2), with insignificant factors dropped from the final modelled response. The predicted maximum response and optimized conditions for each analyte were then used to determine the final global optimized sample incubation time, fiber exposure time, and sample volumes at each ethanol concentration. This was completed according to previously published methods [22] in which a desirability function is used to maximize the sampling sensitivity for analytes with low detector responses within the calibration range (presented separately for 5, 8 and 13% *v*/*v* ethanol concentration in the Supplementary Tables S3–S5, respectively). All statistical testing and modeling were conducted in Matlab R2007a (The Mathworks, Natick, MA, USA).

### 2.5. Calibration Curves

Calibration curves were obtained by diluting the highest concentration mixture of pure compounds to eight concentrations over the analytical range. Calibration samples of different concentrations were prepared for the 5, 8 and 13% *v*/*v* ethanol content model solutions and the samples were injected into the GC-MS instrument according to the optimized exposure time, temperature, and volume of the three different ethanol content solutions. The calibration curves for the compounds of interest at three different ethanol concentrations were prepared using the ChemStation^®^ (Agilent Technologies, Ltd.) software. The linear regression equation for each compound was determined from the calibration curve of the peak area ratio (volatile compound/internal standard) versus the compound concentrations in the solution. The limit of the quantification (LOQ) was evaluated from the determination of the signal to noise ratio for all compounds with a minimum ratio of 10 used as the criteria.

### 2.6. Natural and Reduced Alcohol Wines

Shiraz and Chardonnay wines were supplied by the Charles Sturt University’s winery and contained 13.7 and 12.2% *v*/*v* ethanol, respectively. Both wines were produced according to conventional winemaking protocols, which included oak maturation for between 6–10 months in accordance with the commercial wine styles. Two reduced alcohol wines containing 8 and 5% *v*/*v* ethanol were produced from both Shiraz and Chardonnay wines. The ethanol content was measured using a near infrared spectrophotometer (Anton Paar Alcoholyzer Graz, Austria). Partial dealcoholization was performed using a bench-top laboratory membrane filtration system (Memstar AA MEM-066 Oakleigh, Australia), which consisted of a reverse osmosis (RO) unit followed by evaporative perstraction (EP), as previously described [25]. Briefly, this two-stage filtration process ensures that the wine is first separated by reverse osmosis into the retentate (concentrate; alcohol reduced) and permeate (filtrate; alcohol enriched) streams. The permeate and stripping solution (water) counter-flowed on either side of the membrane contactor while the alcohol is discharged by means of perstraction (evaporation and diffusion) through the membrane. The dealcoholized permeate is then cooled and recombined with the feed wine. Prior to bottling into dark green 750 mL glass bottles, the concentration of molecular sulfur dioxide of all samples was adjusted to 0.5–0.8 mg/L. The bottles were screw-capped and stored at 15 °C until further analysis.

### 2.7. Reconstituted Wines

Reconstituted wines, consisting of 5% *v*/*v* reduced alcohol wines adjusted back to their natural ethanol content, were prepared by adding 8.7 mL ethanol (VWR Prolabo, Selangor, Malaysia) per 100 mL of Shiraz wine, and 7.2 mL ethanol per 100 mL of Chardonnay. Prior to bottling into dark green 750 mL glass bottles, the concentration of molecular sulfur dioxide was adjusted to give 0.5–0.8 mg/L. The bottles were screw-capped and stored at 1.8 °C for 24 h and 14 days until the HS-SPME GC-MS analysis. The reconstituted wines allow us to differentiate the effect of partial dealcoholization by the RO-EP treatment from the effect of the ethanol content reduction on the wine volatile composition. The times chosen allowed for the assessment of any immediate impacts on the volatile profile (24 h), and any effect after the time required to permit ethanol integration (14 days).

### 2.8. GC-MS Analysis of Wine Samples

Samples (10 mL) of reduced alcohol treatments (i.e., 5 and 8% *v*/*v*), as well as those (8 mL) of alcoholized ones (i.e., natural and reconstituted), were accurately pipetted into 20 mL SPME vials. The internal standard mix (100 µL) was added to each vial. All vials were screw-capped, cooled at 8 °C, and moved to the heater block at 45 °C prior to allowing equilibration for 20 min while shaken (250 rpm). The incubation temperatures of 5, 8 and 13% *v*/*v* ethanol content samples were 48, 46 and 44 °C respectively. A DVB/CAR/PDMS SPME fiber was exposed into the sample vial headspace. The extraction times of volatile compounds for the 5, 8, and 13% *v*/*v* samples were 29, 43, and 42 min respectively. Extraction was performed with vial shaking and the extracted sample was inserted into the GC-MS injector at 260 °C in the splitless mode for 1 min.

## 3. Results and Discussion

### 3.1. Optimization of SPME Factors

Optimization of SPME sample experimental conditions is a crucial analytical step to ensure the accuracy and sensitivity of the method for headspace analysis [18]. In the present study, a quantitation method for a range of wine impact odorants was optimized by a three-level Box-Behnken design for three different ethanol content wine-model solutions (i.e., 5, 8 and 13% *v*/*v*). This multivariate statistical model minimized the number of experiments required to determine the significant coefficients for linear interactions and quadratic terms for SPME fiber exposure temperature, time, and wine sample volume for each analyte responses. A global optimized condition for SPME sampling for the suite of analytes, at each ethanol concentration was then developed by using a desirability function that accounts for a low signal response for compounds. This approach has been previously employed for determining the optimized conditions for the quantification of fungal off-flavors [22].

Global optimized SPME sampling conditions (at each ethanol concentration) were determined from the predicted maximum response for each analyte and associated sampling conditions (Equation (2) and Tables S3–S5) using the *b*-coefficients, which were determined using the design matrix (Supplementary Materials) and measured responses. A global and weighted mean SPME fiber extraction temperature, time, and sample volume are determined from the relative size of the inverse maximum predicted response. This approach increases the importance of maximizing the SPME sampling conditions for compounds with relatively low detector responses, whilst down weighting the influence of compounds with large detector responses.

Ethanol is the most abundant volatile compound in wine and will compete with other compounds of interest for binding sites on the SPME fiber [26]. As wine ethanol concentrations impact the partitioning of volatile compounds from liquid to the headspace, optimized sample analysis conditions were determined at wine ethanol levels to avoid confounding analytical results associated with sample dilution. This approach ensures that the headspace concentrations for each compound of interest at each ethanol concentration are accurately determined and allow for comparisons between wines at differing ethanol levels to be made. The results regarding the combination of optimized fiber exposure time, temperature, and sample volume at different ethanol contents are summarized in Supplementary Materials (Tables S3–S5 for 5, 8 and 13% *v*/*v* ethanol content model wine solutions, respectively).

**Figure 1:**
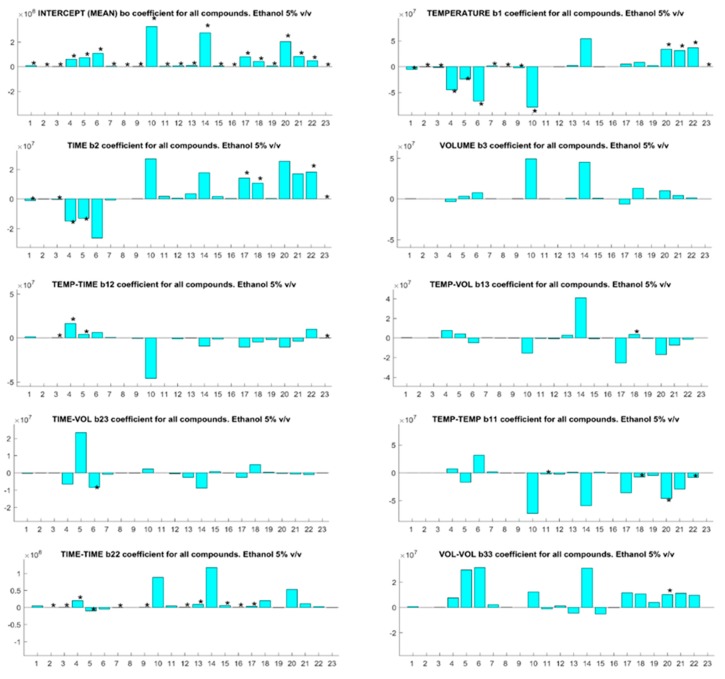
The *b*-coefficients (absolute units) for solid phase microextraction (SPME) optimization parameters for 23 compounds with an ethanol concentration of 5% *v*/*v*. Significant coefficients for specific compounds are indicated with an asterisk (*). Compound identification is cross-referenced to the numbered compounds in Table 1.

**Figure 2:**
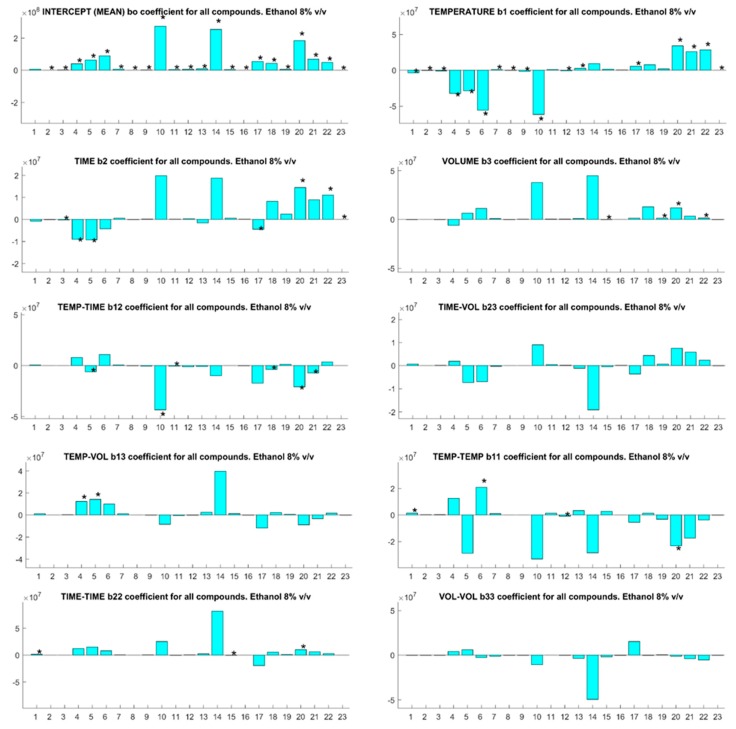
The *b*-coefficients (absolute units) for SPME optimization parameters for 23 compounds with an ethanol concentration of 8% *v*/*v*. Significant coefficients for compounds are indicated with an asterisk (*). Compound identification is cross-referenced to the numbered compounds in Table 1.

**Figure 3:**
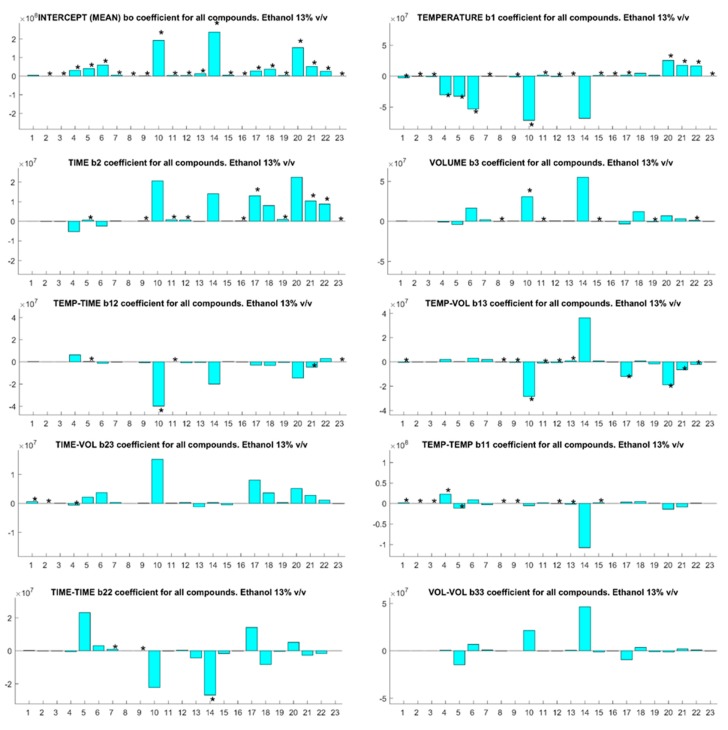
The *b*-coefficients (absolute units) for SPME optimization parameters for 23 compounds with an ethanol concentration of 13% *v*/*v*. Significant coefficients for specific compounds are indicated with an asterisk (*). Compound identification is cross-referenced to the numbered compounds in Table 1.

Inspection of the *b*-coefficients for each compound at each ethanol concentration (Figure 1, Figure 2 and Figure 3 for 5, 8, and 13% *v*/*v* ethanol content model wine solutions) reveals the impact of each experimental factor upon the analyte behavior for SPME. The modulus of each *b*-coefficient is determined by the relative detector response for each analyte and the sign infers either a positive or negative impact upon the analytical sensitivity. The SPME temperature of fiber exposure is the most critical factor for analyte quantification in agreement with the previous findings for different matrices other than wine [27,28]. This is indicated by the number of analytes with a significant *b*-coefficient for the linear and second-order interaction terms of temperature, and this trend is evident at all ethanol concentrations. Of interest is the association of the negative *b*-coefficients of temperature with early eluting compounds (1–10) and the positive *b*-coefficients of late eluting compounds (15–23), and this can be correlated with the boiling point and the elution order of the analytes of interest (Supplementary Table S6). The overall SPME fiber binding of analytes from the vial headspace is determined by mass transfer of the analytes from the liquid to volatile phase followed by mass transfer onto the fiber. Temperature has a significant impact upon the vapor pressures of analytes and, therefore, upon the headspace concentrations in which the SPME fiber is immersed during sampling [29]. Increasing the sample temperature will inevitably increase the headspace concentrations of the higher boiling point analytes, leading to improved analytical sensitivities for these compounds and increasing the competitive binding for SPME fiber space with lower boiling point analytes [13]. Increased competition between high and low boiling point compounds for fiber space explains the negative *b*-coefficients for early eluting compounds.

SPME fiber exposure time is important for the quantification of compounds. Negative *b*-coefficient for linear terms are associated with early eluting compounds and positive coefficients for late eluting and high boiling point compounds, which is evident at all ethanol concentrations. The partitioning of volatile compounds into the headspace during fiber exposure is a dynamic phenomenon as the compounds absorb onto the SPME fiber from the headspace [30]. As semivolatile compound concentrations in the headspace are relatively low compared to their liquid concentration, the overall mass transfer rates are low and the longer extraction times lead to the increased mass transfer of these compounds from the vapor onto the fiber.

Interestingly, the sample volume in the headspace vial was insignificant for all compounds at 5% *v*/*v* ethanol (Figure 1), but became a significant factor for some compounds as the ethanol concentration increased (Figure 2 and Figure 3). This observation should be considered in light of the relative concentration and partition coefficients of ethanol in the base wines compared to the analytes of interest. As the ratio of sample to headspace volume increases, compounds with high partition coefficients—i.e., compounds with relatively lower headspace concentrations—partition less into the headspace relative to compounds with low partition values. Ethanol has a relatively high partition coefficient but is present in high concentrations relative to other volatile compounds. Competition between ethanol and other volatile compounds for SPME fiber binding sites will arise, which would lower sampling sensitivity. Larger sample volumes will increase the quantity of some analytes present in the headspace vial relative to ethanol, thereby, increasing the sensitivity of the SPME sampling for these compounds at higher ethanol concentrations.

The calibration parameters, including analyte quantification and qualification ions, elution times and method performance characteristics, are presented in Supplementary Table S6. The linearity, limits of quantification, and signal to noise ratios are within the acceptable thresholds for compound quantification [21].

### 3.2. Volatile Changes after RO-EP Treatment

Table 3 and Table 4 report the results of the GC-MS analysis of all analytes determined in the natural and reduced alcohol wines for Shiraz and Chardonnay, respectively. A total of 23 compounds were identified and quantified in the different headspace extracts, namely, esters (13), alcohols (3), acids (3), terpenes (2), sulfur compounds (1), and phenols (1). Analysis of variance (ANOVA) showed that the concentration of most analytes clearly decreased with dealcoholization by up to 82% from their natural concentration.

As reported in Table 3 and Table 4, most esters decreased in both wine varieties by 20–81% from their natural concentration, except for methyl octanoate and methyl decanoate in both wine varieties at all dealcoholized levels, and isoamyl octanoate and ethyl-s-lactate in the Shiraz and Chardonnay wines, respectively. The percentage losses of esters increased with the increased extent of dealcoholization (from 8 to 5% *v*/*v*). For example, ethyl-3-methyl butyrate and isoamyl acetate were not appreciably diminished in the 8% *v*/*v* Shiraz and Chardonnay treatments in comparison to the natural wines, but they decreased in the 5% *v*/*v* samples.

Three alcohols (other than ethanol) have been identified and quantified in the wines, originating from grape-derived precursors, such as (z)-3-hexenol, or yeast’s metabolism, such as 3-methyl-1-butanol and β-phenyl ethanol. It is noteworthy that these alcohols followed a similar trend for both wine varieties. Whereas 3-methyl-1-butanol and (z)-3-hexenol decreased with dealcoholization by 32–41% and 13–21% for Shiraz and Chardonnay respectively; no differences were found between 5 and 8% *v*/*v* ethanol wines.

Another additional piece of information is that β-phenyl ethanol, which has a characteristic odor of rose-honey-like at a high concentration of ≥14,000 µg/L [31], significantly increased with dealcoholization by up to 25% and 44% in the Shiraz and Chardonnay wines, respectively. The greatest increase for β-phenyl ethanol in the Shiraz and Chardonnay wines was observed in the 8% *v*/*v* treatment compared to the standard wines. For this compound, a retention effect exerted by the wine non-volatile matrix, possibly due to the π-π stacking interactions with wine polyphenols, was previously suggested [14,25].

For both wine varieties, propanoic acid did not decrease at any dealcoholization level. On the other hand, octanoic acid decreased by up to 21% and 14% in the Shiraz and Chardonnay wines, respectively. These results are in general agreement with the previous studies for similar membrane contactors, in which losses of up to 17% and 68% for octanoic acid and decanoic acid were respectively reported [32]. Of all quantified acids, decanoic acid was the most decreased. It decreased by up to 58% and 77% in the Shiraz and Chardonnay wines, respectively. Nevertheless, no differences were observed between the 8 and 5% *v*/*v* treatments for both wine varieties.

Two monoterpenes were identified and quantified in the Shiraz and Chardonnay wines, namely linalool and geraniol. Intriguingly, the concentration of linalool, which has been reported to have a characteristic lavender flower aroma [31], increased by up to 58% in the Shiraz dealcoholized wines. This was in contrast to the Chardonnay samples where the concentration of linalool decreased with dealcoholization by up to 78% from its natural concentration, possibly due to the different non-volatile matrix composition between these wines [33]. Whereas a reduced concentration in geraniol of 70–82% is perhaps not surprising for Chardonnay wines, a loss of geraniol of 64% in Shiraz dealcoholized samples is apparently in contrast with the previously suggested retention effect from non-volatile wine compounds towards aroma compounds. This may be due to the differences in the type of monoterpenes, suggesting the important effect of the molecular chemical structure in the interaction with some non-volatile compounds [33].

The concentration of other compounds such as 3-(methylthio)-1-propanol did not change at any dealcoholization extent for both wine varieties. This sulfur-containing aroma compound, principally arising from the alcoholic fermentation, can impart an undesirable raw potato odor at a high concentration of ≥1000 µg/L [31]. Likewise, vanillin was not significantly decreased by the dealcoholization treatment in both the Shiraz and Chardonnay wine varieties. 

### 3.3. Effect of Ethanol Content Reduction

The overall effect of dealcoholization by RO-EP treatment on the wine’s volatile composition includes two distinct effects: (i) the RO-EP effect and (ii) the ethanol content reduction effect [7]. To quantify the effect arising from the ethanol content reduction, a comparison between the reconstituted and 5% dealcoholized wines, was carried out. GC-MS analyses on reconstituted samples were performed after 24 h and 14 days of storage at 1.8 °C. If the ethanol content had either a suppressing or enhancing the effect on analyte responses, the addition of exogenous ethanol to the 5% *v*/*v* dealcoholized samples should result in a significant difference between the GC-MS results of reconstituted and 5% reduced alcohol wines.

As indicated by Table 3 and Table 4 for the Shiraz and Chardonnay wines, respectively, the concentration of many analytes was lower in the reconstituted wines compared to that of the 5% *v*/*v* ethanol samples. These values must be viewed in terms of the minor dilution of around 7–8% arising from the ethanol addition to reconstitute the wine to the original ethanol concentration. The concentration of ethyl decanoate in the reconstituted Shiraz and Chardonnay wines significantly decreased after 24 h storage by 88% and 79%, respectively, compared to the corresponding 5% *v*/*v* ethanol samples. ANOVA, however, shows that the number of volatile compounds that decreased in the headspace of reconstituted wines was different between the Chardonnay and Shiraz wine varieties, and slightly higher after 24 h (10/23 and 12/23 for the Shiraz and Chardonnay wines, respectively) than after 14 days (8/23 and 11/23 for the Shiraz and Chardonnay wines, respectively) storage. A negative relationship between ethanol and other volatile compounds is consistent with the previous studies for other wines or model solutions [15,16,34]. Differences in the number of affected analytes between Shiraz and Chardonnay wine varieties likely arise from a different composition in wine non-volatile matrix, including an expected higher presence of phenolics in red than in white wine.

While several volatile compounds exhibited a negative relationship with ethanol, many others did not show any appreciable interaction. For example, the concentration of ethyl butyrate, ethyl-2-methyl butyrate, and ethyl-3-methyl butyrate did not change at any storage extent (i.e., 24 h, 14 days) for both Shiraz and Chardonnay reconstituted wines compared to the 5% *v*/*v* ethanol samples. In the case of vanillin only, its concentration in the wine headspace of the reconstituted Shiraz and Chardonnay wines increased after 24 h storage by 60% and 72%, respectively. However, no differences were observed after 14 days of storage.

Overall, these results indicate that the reduction of the ethanol content in the Shiraz and Chardonnay wines significantly affected the headspace composition of wine, however, the nature of changes on the different classes of analytes is not specific.

## 4. Conclusions

A total of 23 compounds extracted using a DVB/CAR/PDMS SPME fiber were successfully quantified using GC-MS. The optimum HS-SPME conditions for the extraction of 23 target impact odorants in reduced alcohol red and white wine that contributed to the regression models using a Box-Behnken experimental design were determined in order to study the effect of the SPME conditions, namely, extraction time, extraction temperature, and sample volume. The three-factor Box-Behnken analysis showed significant (*p* < 0.05) relationships between the SPME variables and the component headspace concentrations, with the extraction temperature resulting in the most critical factor when studying the equilibrium of reduced alcohol wine’s impact odorants.

The concentration of several analytes decreased with dealcoholization, with ethyl hexanoate and ethyl octanoate being the most affected. It was also shown that the concentration of many volatile compounds remained significantly decreased in the headspace of reconstituted wines (i.e., 5% *v*/*v* reduced alcohol wines adjusted back to their natural ethanol concentration) compared to that of 5% *v*/*v* reduced alcohol samples, confirming losses of these compounds from the wine during the ethanol removal process and revealing that the final composition of the reduced alcohol wine’s headspace is mainly due to the combination of two factors: (i) the type of the dealcoholization treatment and (ii) the ethanol content reduction.

A negative relationship between the ethanol concentration and most analyte responses during the SPME sampling process is apparent and the confounding impact of competition for SPME binding sites between ethanol and other volatile compounds requires a rigorous analytical approach to enable comparisons between wines of varying ethanol concentrations. Sample dilution to a constant ethanol concentration will alleviate the inconsistent competition between ethanol and analyte binding on the SPME fiber but will also alter the partitioning of compounds from the liquid to headspace, thereby confounding the final comparisons of different wines. Researchers should consider that variations in sample conditions such as the ethanol content may confound comparisons of different wines, particularly those between natural and dealcoholized treatments. For the most accurate assessment of the concentration of volatile compounds, analytical conditions that account for varying ethanol concentrations must be used. Invariably, this will require analytical calibrations at each important ethanol concentration for headspace analysis.

## Figures and Tables

**Table 1:** The compound identification for Box-Behnken design, odors and olfactory thresholds, and boiling point.

Code	Compounds	Odors ^1^	OT ^1^ (µg/L)	BP (°C)
1	ethyl butyrate	apple	20	121
2	ethyl-2-methyl butyrate	apple	1–18	138
3	ethyl-3-methyl butyrate	sweat, acid, rancid	33.4	134
4	isoamyl acetate	banana	30	130
5	3-methyl-1-butanol	whiskey, malt, burnt	30,000	132
6	ethyl hexanoate	apple peel, fruit	2–14	167
7	ethyl-s-lactate	fruit, milk ^2^	154,000	154
8	(z)-3-hexenol	green (cut grass)	400	156
9	methyl octanoate	waxy, apple peel ^2^	-	192
10	ethyl octanoate	fruit, fat	2–5	207
11	propanoic acid	pungent, rancid, soy	8100	141
12	linalool	flower, lavender	25.2	198
13	methyl decanoate	wax, soap, fruit ^2^	-	108
14	ethyl decanoate	grape	200	245
15	isoamyl octanoate	wax, soap, pear ^2^	-	267
16	3-(methylthio)-1-propanol	sweet, potato	1000	90
17	β-phenyl ethyl acetate	rose, honey	250	229
18	ethyl dodecanoate	wax, soap ^2^	-	269
19	geraniol	rose, geranium	30	230
20	β-phenyl ethanol	honey, rose	10,000–14,000	219
21	octanoic acid	sweat, cheese	500	240
22	decanoic acid	rancid, fat	1000	268
23	vanillin	vanilla	200	285

OT: odor threshold; BP: boiling point. ^1^ From Francis and Newton [20] except where specified. ^2^ From Antalick, et al. [21].

**Table 2:** The Box-Behnken (three-factor) design.

Parameter/Conditions	Levels
Extraction temperature (°C)	30, 50, 70
Extraction time (min)	15, 30, 45
Sample volume (mL) in 20 mL vial	7, 10, 13

**Table 3:** The changes in the headspace concentration of volatile compounds in Shiraz wines.

Compound (µg L^−1^)	Dealcoholized and Natural Wines			Reconstituted Wines	
	5% *v*/*v*	8% *v*/*v*	13.7% *v*/*v*	Storage Time	
				24 h	14 Days
ethyl butyrate	0.90 ± 0.01 ^a^	1.72 ± 0.08 ^b^	2.69 ± 0.06 ^c^	0.75 ± 0.01 ^a^	0.76 ± 0.02 ^a^
ethyl-2-methyl butyrate	0.33 ± 0.00 ^b^	0.46 ± 0.03 ^c^	0.47 ± 0.01 ^c^	0.23 ± 0.01 ^a^	0.17 ± 0.00 ^a^
ethyl-3-methyl butyrate	0.05 ± 0.00 ^b^	0.08 ± 0.01 ^c^	0.08 ± 0.00 ^c^	0.04 ± 0.00 ^ab^	0.03 ± 0.00 ^a^
isoamyl acetate	0.21 ± 0.01 ^b^	0.43 ± 0.03 ^c^	0.51 ± 0.03 ^c^	0.08 ± 0.01 ^a^	0.08 ± 0.01 ^a^
3-methyl-1-butanol	6.18 ± 0.06 ^ab^	6.64 ± 0.41 ^b^	9.05 ± 0.24 ^c^	5.62 ± 0.17 ^ab^	5.38 ± 0.13 ^a^
ethyl hexanoate	1.72 ± 0.02 ^a^	2.34 ± 0.23 ^a^	5.41 ± 0.88 ^b^	1.38 ± 0.11 ^a^	1.51 ± 0.05 ^a^
ethyl-s-lactate	33,500 ± 720 ^a^	32,300 ± 2600 ^a^	47,300 ± 3060 ^b^	41,900 ± 1870 ^ab^	33,900 ± 1560 ^a^
(z)-3-hexenol	5.32 ± 0.10 ^c^	5.42 ± 0.04 ^c^	6.83 ± 0.05 ^d^	3.61 ± 0.14 ^a^	4.49 ± 0.08 ^b^
methyl octanoate	0.01 ± 0.00	0.01 ± 0.00	0.02 ± 0.00	BLQ	BLQ
ethyl octanoate	3.17 ± 0.01 ^b^	4.90 ± 0.15 ^c^	7.32 ± 0.15 ^d^	2.31 ± 0.02 ^a^	2.17 ± 0.05 ^a^
propanoic acid	600 ± 20 ^c^	250 ± 10 ^ab^	220 ± 0.00 ^a^	240 ± 0.00 ^a^	290 ± 10 ^b^
linalool	0.53 ± 0 ^b^	0.59 ± 0.01 ^b^	0.25 ± 0.06 ^a^	0.27 ± 0.01 ^a^	0.27 ± 0.00 ^a^
methyl decanoate	BLQ	0.01 ± 0.00	0.01 ± 0.00	0.01 ± 0.00	0.01 ± 0.00
ethyl decanoate	1.66 ± 0.02 ^bc^	1.39 ± 0.10 ^b^	2.64 ± 0.46 ^c^	0.19 ± 0.13 ^a^	0.74 ± 0.09 ^ab^
isoamyl octanoate	0.02 ± 0.00 ^b^	0.02 ± 0.00 ^b^	0.02 ± 0.00 ^b^	0.01 ± 0.00 ^a^	0.01 ± 0.00 ^a^
3-(methylthio)-1-propanol	103 ± 0.42 ^a^	101 ± 0.87 ^a^	103 ± 12.0 ^a^	92.1 ± 3.44 ^a^	90.7 ± 9.40 ^a^
β-phenyl ethyl acetate	0.03 ± 0.00 ^a^	0.04 ± 0.00 ^a^	0.05 ± 0.00 ^b^	0.02 ± 0.00 ^a^	0.03 ± 0.00 ^a^
ethyl dodecanoate	0.04 ± 0.00 ^a^	0.03 ± 0.00 ^a^	0.12 ± 0.01 ^b^	0.02 ± 0.00 ^a^	0.02 ± 0.00 ^a^
geraniol	0.09 ± 0.00 ^a^	0.09 ± 0.00 ^a^	0.25 ± 0.03 ^b^	0.06 ± 0.00 ^a^	0.06 ± 0.00 ^a^
β-phenyl ethanol	280 ± 0.00 ^d^	320 ± 0.00 ^e^	240 ± 0.00 ^c^	230 ± 0.00 ^b^	200 ± 0.00 ^a^
octanoic acid	8.00 ± 0.12 ^c^	7.10 ± 0.19 ^b^	9.01 ± 0.3 ^d^	6.58 ± 0.08 ^b^	5.36 ± 0.10 ^a^
decanoic acid	0.94 ± 0.01 ^a^	0.90 ± 0.02 ^a^	2.16 ± 0.22 ^b^	1.16 ± 0.05 ^a^	0.96 ± 0.05 ^a^
vanillin	0.80 ± 0.06 ^a^	1.16 ± 0.1 ^ab^	1.13 ± 0.09 ^ab^	2.01 ± 0.40 ^b^	0.92 ± 0.08 ^a^

Data are expressed as the mean of triplicate determinations ± standard deviation. Different letters in a row discriminate the treatments that are significantly different from one another (*p* ≤ 0.05). BLQ: below limit of quantitation. Values for reconstituted wines are not corrected for dilution by ethanol addition (~7%).

**Table 4:** The changes in the headspace concentration of volatile compounds in Chardonnay wines.

Compound (µg L^−1^)	Dealcoholized and Natural Wines			Reconstituted Wines	
	5% *v*/*v*	8% *v*/*v*	12.2% *v*/*v*	Storage Time	
				24 h	14 Days
ethyl butyrate	0.96 ± 0.01 ^a^	2.42 ± 0.23 ^b^	4.12 ± 0.05 ^c^	0.87 ± 0.02 ^a^	0.88 ± 0.01 ^a^
ethyl-2-methyl butyrate	0.11 ± 0.00 ^b^	0.20 ± 0.01 ^c^	0.25 ± 0.00 ^d^	0.09 ± 0.00 ^ab^	0.07 ± 0.00 ^a^
ethyl-3-methyl butyrate	0.02 ± 0.00 ^a^	0.04 ± 0.01 ^b^	0.05 ± 0.00 ^b^	0.02 ± 0.00 ^a^	0.01 ± 0.00 ^a^
isoamyl acetate	0.04 ± 0.01 ^a^	0.23 ± 0.01 ^b^	0.21 ± 0.00 ^b^	BLQ	BLQ
3-methyl-1-butanol	3.92 ± 0.04 ^ab^	5.56 ± 0.26 ^bc^	6.68 ± 0.74 ^c^	2.78 ± 0.11 ^a^	2.92 ± 0.07 ^a^
ethyl hexanoate	3.79 ± 0.04 ^a^	5.77 ± 0.70 ^b^	11.6 ± 0.33 ^c^	2.62 ± 0.12 ^a^	2.84 ± 0.03 ^a^
ethyl-s-lactate	4790 ± 80 ^ab^	5230 ± 250 ^ab^	6560 ± 930 ^b^	4360 ± 260 ^a^	4280 ± 130 ^a^
(z)-3-hexenol	3.75 ± 0.06 ^c^	4.08 ± 0.03 ^c^	4.68 ± 0.14 ^d^	2.35 ± 0.07 ^a^	3.07 ± 0.03 ^b^
methyl octanoate	0.01 ± 0.00	0.01 ± 0.00	0.03 ± 0.00	BLQ	BLQ
ethyl octanoate	8.82 ± 0.09 ^b^	15.5 ± 0.71 ^c^	32.3 ± 0.65 ^d^	5.23 ± 0.02 ^a^	5.82 ± 0.07 ^a^
propanoic acid	200 ± 20	230 ± 10	180 ± 0.00	180 ± 0.00	210 ± 0.00
linalool	0.29 ± 0.00 ^c^	0.37 ± 0.00 ^d^	0.08 ± 0.00 ^b^	0.05 ± 0.00 ^a^	0.06 ± 0.00 ^a^
methyl decanoate	BLQ	0.01 ± 0.00	0.02 ± 0.00	0.01 ± 0.00	0.01 ± 0.00
ethyl decanoate	4.18 ± 0.06 ^b^	3.59 ± 0.31 ^b^	12.4 ± 0.33 ^c^	0.87 ± 0.42 ^a^	1.82 ± 0.03 ^a^
isoamyl octanoate	0.04 ± 0.00 ^b^	0.03 ± 0.00 ^b^	0.06 ± 0.00 ^c^	0.01 ± 0.00 ^a^	0.01 ± 0.00 ^a^
3-(methylthio)-1-propanol	40.9 ± 0.22 ^b^	43.3 ± 1.76 ^b^	38.2 ± 1.46 ^b^	29.1 ± 1.61 ^a^	40.4 ± 1.27 ^b^
β-phenyl ethyl acetate	0.03 ± 0.00 ^ab^	0.04 ± 0.00 ^bc^	0.04 ± 0.00 ^c^	0.03 ± 0.00 ^a^	0.03 ± 0.00 ^a^
ethyl dodecanoate	0.06 ± 0.00 ^b^	0.07 ± 0.00 ^b^	0.3 ± 0.01 ^c^	0.02 ± 0.00 ^a^	0.02 ± 0.00 ^a^
geraniol	0.20 ± 0.00 ^c^	0.12 ± 0.00 ^b^	0.67 ± 0.01 ^d^	0.14 ± 0.02 ^b^	0.06 ± 0.00 ^a^
β-phenyl ethanol	90 ± 0.00 ^b^	90 ± 0.00 ^b^	50 ± 0.00 ^a^	50 ± 0.00 ^a^	50 ± 0.00 ^a^
octanoic acid	30.3 ± 0.14 ^b^	35.7 ± 0.67 ^c^	35.2 ± 1.12 ^c^	24.6 ± 0.12 ^a^	22.1 ± 0.12 ^a^
decanoic acid	3.37 ± 0.04 ^a^	3.72 ± 0.34 ^a^	15.7 ± 0.60 ^b^	3.69 ± 0.16 ^a^	2.61 ± 0.08 ^a^
vanillin	0.08 ± 0.01 ^a^	0.12 ± 0.01 ^a^	0.19 ± 0.01 ^ab^	0.29 ± 0.05 ^b^	0.15 ± 0.02 ^a^

Data are expressed as the mean of triplicate determinations ± standard deviation. Different letters in a row discriminate the treatments that are significantly different from one another (*p* ≤ 0.05). BLQ: below limit of quantitation. Values for reconstituted wines are not corrected for dilution by ethanol addition (~7%).

## Supplementary Materials

The following are available online at http://www.mdpi.com/2304-8158/7/8/127/s1, Table S1: Design matrix for the optimization experiment using experimental conditions; Table S2: Coded design matrix where experimental factors are coded −1, 0, 1 at each level; Table S3: Optimized SPME conditions for target aroma compounds for the 5% *v*/*v* treatment; Table S4: Optimized SPME conditions for target aroma compounds for the 8% *v*/*v* treatment; Table S5: Optimized SPME conditions for target aroma compounds for the 13% *v*/*v* treatment; Table S6: Target compounds identification and calibration parameters for three different levels of ethanol content of wine.
