# Supplementary material for: SPME Method Optimized by Box-Behnken Design for Impact Odorants in Reduced Alcohol Wines
Source: Foods. 2018 Aug 10;7(8):127. doi: 10.3390/foods7080127 (PMC6112000; doi:10.3390/foods7080127)
Supplement: Supplementary file 1 [file foods-07-00127-s001.pdf]

foods

Supplementary Material

Optimization of SPME conditions for each analyte response was undertaken using a Box-Behnken design of experiments approach with statistical testing based upon Brereton, 2003. A series of randomized experiments with extraction conditions set to test each parameter combination in Table 1 was performed. A feature of this design is the center point for each experimental factor is equidistant to the extremes. Each analyte response at each set of conditions is then used to determine a predictive response based upon the following equation:

$$\hat{y} = b_0 + b_1x_1 + b_2x_2 + b_3x_3 + b_{12}x_1x_2 + b_{13}x_1x_3 + b_{23}x_2x_3 + b_{11}x_1^2 + b_{22}x_2^2 + b_{33}x_3^2$$

where

$\hat{y}$ = predicted response

b_0 = intercept or average response

$b_1x_1 + b_2x_2 + b_3x_3$ = linear terms associated with each factor (temp, time, sample vol.)

$b_{12}x_1x_2 + b_{13}x_1x_3 + b_{23}x_2x_3$ = second order interaction terms between each factor

$b_{11}x_1^2 + b_{22}x_2^2 + b_{33}x_3^2$ = quadratic terms for each factor

x_1 = factor extraction temperature

x_2 = factor extraction time

x_3 = factor sample volume in 20 mL vial

The relationship between an analyte response, the b coefficients and the experimental conditions can be expressed in a matrix form as:

$\hat{y} = D.b$

where D = the design matrix

A design matrix for the optimization experiment can be constructed from the experimental conditions.

Table S1. Design Matrix for SPME Optimization with central conditions replicated.

	Linear Terms			Second Order Interactions			Quadratic Terms		
*b*0	*b*1	*b*2	*b*3	*b*12	*b*13	*b*23	*b*11	*b*22	*b*33
Intercept	Temperature	Time	Volume	Temp x Time	Temp x Vol	Time x Vol	Temp x Temp	Time x Time	Vol x Vol
1	30	15	7	450	210	105	900	225	49
1	30	15	13	450	390	195	900	225	169
1	30	30	10	900	300	300	900	900	100
1	30	45	7	1350	210	315	900	2025	49
1	30	45	13	1350	390	585	900	2025	169
1	50	15	10	750	500	150	2500	225	100
1	50	30	7	1500	350	210	2500	900	49
1	50	30	10	1500	500	300	2500	900	100
1	50	30	13	1500	650	390	2500	900	169
1	50	45	10	2250	500	450	2500	2025	100
1	70	15	13	1050	910	195	4900	225	169
1	70	30	10	2100	700	300	4900	900	100
1	70	45	7	3150	490	315	4900	2025	49
1	70	45	13	3150	910	585	4900	2025	169
1	70	15	7	1050	490	105	4900	225	49

To facilitate interpretation of the significance of each b coefficient it is helpful to code the design matrix such that each experimental factor is on a comparable or common scale. Typically, this is achieved by replacing each experimental factor level with *-1, 0* or *1* in the design matrix.

A suitable design matrix for the optimization experiment can be constructed from the experimental conditions.

Table S2. Design Matrix for SPME Optimization with central conditions replicated with experimental factor levels coded by *-1, 0* or *1.*

	Linear Terms			Second Order Interactions			Quadratic Terms		
*b*0	*b*1	*b*2	*b*3	*b*12	*b*13	*b*23	*b*11	*b*22	*b*33
Intercept	Temperature	Time	Volume	Temp x Time	Temp x Vol	Time x Vol	Temp x Temp	Time x Time	Vol x Vol
1	-1	-1	-1	1	1	1	1	1	1
1	-1	-1	1	1	-1	-1	1	1	1
1	-1	0	0	0	0	0	1	0	0
1	-1	1	-1	-1	1	-1	1	1	1
1	-1	1	1	-1	-1	1	1	1	1
1	0	-1	0	0	0	0	0	1	0
1	0	0	-1	0	0	0	0	0	1
1	0	0	0	0	0	0	0	0	0
1	0	0	1	0	0	0	0	0	1
1	0	1	0	0	0	0	0	1	0
1	1	-1	1	-1	1	-1	1	1	1
1	1	0	0	0	0	0	1	0	0
1	1	1	-1	1	-1	-1	1	1	1
1	1	1	1	1	1	1	1	1	1
1	1	-1	-1	-1	-1	1	1	1	1

As the design matrix is not square, i.e. a greater number of experiments than parameter values to be determined, a pseudoinverse must be used to determine b coefficients:

$$b = (D'.D)^{-1}.D'.y$$

Once b coefficients have been calculated for each experimental factor it is possible to predict ŷ and then determine the sum of squares from residuals:

$$SS\_red. = \Sigma\ (y-\hat{y})^2$$

The mean error sum of squares is determined from the sums of squares of the residuals and degrees of freedom such:

$ss\_mean = SS\_red\ /\ (N - P)$ where N = total number of experiments and P = number of coefficients

Variance associated with the b coefficients is derived from the diagonal of the pseudoinverse design matrix:

$$b\_var = \text{diagonal}\ (D'.D)^{-1}$$

Student's t-test can now be used to determine significance for each b coefficient with comparison to the 2 tailed distribution:

$$t_b = b\ /\ \sqrt{(ss\_mean \times b\_var)}$$

52 **Table S3.** Optimized SPME conditions for target aroma compounds for the 5% v/v treatment.

Compound	Temperature (°C)	Extraction Time (min)	Sample Volume (mL)	Predicted Response	Relative Peak Size (RPS)	Inverse RPS	Inverse RPS × OPT Temperature	Inverse RPS × OPT Time	Inverse RPS × OPT Volume
Ethyl butyrate	29.9	14.8	6.94	21645984	0.04	25.8	771	381	179
Ethyl-2-methyl butyrate	29.6	14.8	6.96	2306248	0.00	242	7153	3581	1683
Ethyl-3-methyl butyrate	29.7	14.8	6.96	4408021	0.01	126	3763	1874	881
Isoamyl acetate	29.6	14.8	6.96	172798045	0.31	3.23	95.5	47.8	22.4
3-Methyl-1-butanol	33.7	14.8	7.01	118938289	0.21	4.69	158	69.4	32.9
Ethyl hexanoate	29.6	14.9	13.1	256763255	0.46	2.17	64.2	32.4	28.4
Ethyl-s-lactate	70.4	14.9	13.0	11427110	0.02	48.8	3437	729	636
(z)-3-Hexenol	29.9	14.8	13.1	1703328	0.00	327	9793	4849	4277
Methyl octanoate	29.4	45.2	13.1	7125869	0.01	78.3	2302	3537	1022
Ethyl octanoate	29.7	45.2	13.0	557715584	1.00	1.00	29.7	45.2	13.0
Propanoic acid	70.3	45.2	10.0	8021459	0.01	69.5	4885	3142	696
Linalool	34.2	45.3	13.1	10086563	0.02	55.3	1891	2506	722
Methyl decanoate	70.4	45.3	10.9	21853028	0.04	25.5	1797	1156	277
Ethyl decanoate	55.5	45.2	13.1	464847557	0.83	1.20	76.4	54.2	15.7
Isoamyl octanoate	70.4	45.2	10.0	11602466	0.02	48.1	1430	2172	481
3-(Methylthio)-1-propanol	70.3	45.3	10.6	2184884	0.00	255	17934	11567	2701
β-Phenyl ethyl acetate	59.8	45.2	6.99	92369223	0.17	6.04	361	273	42.2
Ethyl dodecanoate	70.3	45.2	13.1	95472899	0.17	5.84	410	264	76.5
Geraniol	55.9	45.1	13.0	11467404	0.02	48.6	2717	2192	634
β-Phenyl ethanol	50.7	45.2	13.0	276062590	0.49	2.02	102	91.3	26.3
Octanoic acid	61.0	45.3	13.1	120528062	0.22	4.63	282	210	60.4
Decanoic acid	70.3	45.3	13.0	113488948	0.20	4.91	345	223	64.0
Vanillin	70.3	45.3	7.21	509975	0.00	1093	76833	49558	7884
Sum						2480	136632	88554	22457
Weighted mean							55.1	35.7	9.05

Table S4. Optimized SPME conditions for target aroma compounds for the 8% v/v treatment.

Compound	Temperature (°C)	Extraction Time (min)	Sample Volume (mL)	Predicted Response	Relative Peak Size (RPS)	Inverse RPS	Inverse RPS × OPT Temperature	Inverse RPS × OPT Time	Inverse RPS × OPT Volume
Ethyl butyrate	29.9	14.8	6.94	16238803	0.03	29.1	871	431	202
Ethyl-2-methyl butyrate	29.6	14.8	6.96	1831724	0.00	258	764	3825	1798
Ethyl-3-methyl butyrate	29.7	14.8	6.96	3521895	0.01	134	3996	1990	935
Isoamyl acetate	29.6	14.8	6.96	138864602	0.29	3.41	101	50.5	23.7
3-Methyl-1-butanol	33.7	14.8	7.01	105083519	0.22	4.50	152	66.7	31.6
Ethyl hexanoate	29.6	14.9	13.1	212711796	0.45	2.22	65.8	33.2	29.1
Ethyl-s-lactate	70.4	45.2	13.0	10546032	0.02	44.9	3160	2028	585
(z)-3-Hexenol	29.9	45.3	13.1	1288470	0.00	368	10985	16643	4798
Methyl octanoate	29.4	45.2	13.1	5904502	0.01	80.1	2357	3622	1047
Ethyl octanoate	29.7	45.2	13.0	473208764	1.00	1.00	29.7	45.2	13.0
Propanoic acid	70.3	45.2	8.85	7181436	0.01	65.9	4629	2978	583
Linalool	34.2	45.3	13.1	8397224	0.02	56.3	1928	2554	736
Methyl decanoate	70.4	14.6	12.8	16907471	0.04	28.0	1971	407	358
Ethyl decanoate	55.5	45.2	13.1	384592070	0.81	1.23	68.3	55.6	16.1
Isoamyl octanoate	70.4	45.2	10.4	9153205	0.02	51.7	3641	2336	539
3-(Methylthio)-1-propanol	70.3	45.3	10.6	1854344	0.00	255	17928	11564	2701
β-Phenyl ethyl acetate	59.8	45.2	6.99	70705745	0.15	6.69	400	302	46.8
Ethyl dodecanoate	70.3	45.2	13.1	85079298	0.18	5.56	391	251	72.8
Geraniol	53.9	45.1	13.0	9499880	0.02	49.8	2684	2245	650
β-Phenyl ethanol	50.7	45.2	13.0	240643663	0.51	1.97	100.0	88.9	25.6
Octanoic acid	58.2	45.3	13.1	98862998	0.21	4.79	278	217	62.5
Decanoic acid	70.3	45.3	13.0	96606109	0.20	4.90	344	222	63.7
Vanillin	70.3	45.3	6.94	563633	0.00	840	58985	38046	5824
Sum						2297	122706	90002	21141
Weighted mean							53.4	39.1	9.20

Table S5. Optimized SPME conditions for target aroma compounds for the 13% v/v treatment.

Compound	Temperature (°C)	Extraction Time (min)	Sample Volume (mL)	Predicted Response	Relative Peak Size (RPS)	Inverse RPS	Inverse RPS × OPT Temperature	Inverse RPS × OPT Time	Inverse RPS × OPT Volume
Ethyl butyrate	29.9	14.8	6.94	13701954	0.03	33.1	937	464	217
Ethyl-2-methyl butyrate	29.6	14.8	6.96	2589960	0.01	285	4900	2453	1153
Ethyl-3-methyl butyrate	29.7	14.8	6.96	3072342	0.01	146	4154	2068	972
Isoamyl acetate	29.6	14.8	6.96	123238386	0.29	3.46	103	51.6	24.2
3-Methyl-1-butanol	30.4	14.8	7.01	96504230	0.22	4.46	150	65.8	31.2
Ethyl hexanoate	29.6	14.9	13.1	189437593	0.44	2.33	67.0	33.8	29.6
Ethyl-s-lactate	70.4	45.2	13.0	10037916	0.02	41.8	3010	1932	557
(z)-3-Hexenol	29.9	45.3	13.1	1122492	0.00	372	11433	17322	4993
Methyl octanoate	29.4	45.2	13.1	5284504	0.01	82.0	2388	3669	1061
Ethyl octanoate	29.7	45.2	13.0	429071690	1.00	1.00	29.7	45.2	13.0
Propanoic acid	70.3	45.2	7.98	7102412	0.02	49.7	4244	2730	534
Linalool	31.1	45.3	13.1	7676236	0.02	53.3	1736	2533	730
Methyl decanoate	70.4	14.6	12.8	16153172	0.04	21.1	1872	387	340
Ethyl decanoate	43.6	45.2	13.1	351186957	0.82	1.15	62.0	55.2	16.0
Isoamyl octanoate	70.4	14.9	10.0	8463703	0.02	42.9	3570	2291	529
3-(Methylthio)-1-propanol	70.3	45.3	9.71	1761832	0.00	212	17110	11036	2366
β- Phenyl ethyl acetate	59.8	45.2	6.99	60423655	0.14	7.44	425	321	49.6
Ethyl dodecanoate	70.3	45.2	13.1	78999059	0.18	5.36	382	245	71.1
Geraniol	52.9	45.1	13.0	8248338	0.02	59.2	2803	2344	678
β-Phenyl ethanol	48.4	45.2	13.0	223882098	0.52	1.82	95.3	86.6	25.0
Octanoic acid	52.9	45.3	13.1	87838265	0.20	4.99	274	221	63.8
Decanoic acid	70.3	45.3	13.0	82814114	0.19	6.54	364	235	67.4
Vanillin	70.3	45.3	7.21	585904	0.00	636	51450	33186	5080
Sum						2073	111558	83776	19603
Weighted mean							53.8	40.4	9.45

Table S6. Target compounds identification and calibration parameters for three different levels of ethanol content of wine.

Compound	IS	SIM group	Retention time (minutes)	Target ion (m/z)	Qualifier ion (m/z)%	Ethanol (% v/v)	Calibration range (µg/L)	Calibration Equation	r^2	SN ratio at LOQ	RI	Boiling Point (°C)[e]
Ethyl butyrate	IS-2	1	10.23	71	43	5	0.065-1314	y=1.673x+0.1434	0.999	47	1045	121
					88	8		y=1.905x-0.3106	0.992	25	(1048[a])	
					29	13		y=1.879x-0.2368	0.996	24		
Ethyl-2-methyl butyrate	IS-2	1	10.69	57	102	5	0.003-66.3	y=3.708x+0.0161	0.998	16	1061	138
					85	8		y=3.676x+0.0123	0.992	12	(1049[b])	
					29	13		y=3.946x-0.0204	0.998	10		
Ethyl-3-methyl butyrate	IS-2	1	11.14	88	57	5		y=30.34x+0.0533	0.999	10	1076	134
					29	8		y=31.30x+0.0265	0.988	16	(1069[b])	
					85	13		y=33.29x-0.0557	0.997	13		
Isoamyl acetate	IS-1	1	12.61	43	70	5	0.02385-4770	y=49.16x+6.099	0.988	17	1128	130
					55	8		y=65.62x-1.962	0.993	19	(1115[a])	
						13		y=48.76x+7.174	0.967	12		
3-methyl-1-butanol	IS-4	2	15.429	55	70	5	24.359-487180	y=5.546x-1.5090	0.998	55	1230	132
					41	8		y=10.79x-4.539	0.995	49	(1206[d])	
					43	13		y=9.417x-1.477	0.995	61		
Ethyl hexanoate	IS-3	2	15.6	88	99	5	0.1296-2592	y=0.4574x+0.6586	0.972	14	1237	167
					43	8		y=0.7313x+0.2447	0.982	12	(1221[a])	
					70	13		y=0.6915x+0.0808	0.998	20		
Ethyl-s-lactate	IS-4	3	18.72	45	75	5	25-500000	y=0.4980x-0.5463	0.983	27		154
					43	8		y=0.8961x-0.8372	0.991	14	1361	

Compound	IS	SIM group	Retention time (minutes)	Target ion (m/z)	Qualifier ion (m/z)%	Ethanol (% v/v)	Calibration range (μg/L)	Calibration Equation	r^2	SN ratio at LOQ	RI	Boiling Point (°C)[e]
					29	13		y=0.6428x-0.5267	0.977	26		
(z)-3-hexenol	IS-4	3	19.708	67	55	5	0.05325-1065	y=0.0326x-0.0538	0.993	23	1402	156
					39	8		y=0.059x-0.1121	0.982	17	(1407[d])	
					41	13		y=0.0421x-0.0475	0.984	21		
Methyl octanoate	IS-5	3	19.57	74	87	5	0.001-2	y=1.7140x+0.0014	0.996	18	1397	192
					43	8		y=1.785x-0.0025	0.983	13	(1387[d])	
					55	13		y=1.956x-0.0009	0.998	20		
Ethyl octanoate	IS-5	3	20.605	88	101	5	0.041-835	y=0.5172x+0.1990	0.997	31	1442	207
					127	8		y=0.5790x-0.1378	0.995	19	(1433[a])	
					60	13		y=0.6325x+0.0932	0.998	20		
Propanoic acid	IS-4	4	23.11	74	45	5	5.6945-113890	y=0.1603x-0.0166	0.997	18	1556	141.2
					73	8		y=2.4790x-0.5021	0.986	15	(1523d)	
						13		y=1.674x-0.2852	0.982	17		
Linalool	IS-4	4	23.27	71	93	5	0.015-307	y=0.6731x-0.1824	0.998	12	1564	198
					55	8		y=1.144x-0.4038	0.995	16	(1560[b])	
					41	13		y=0.8213x-0.0324	0.996	15		
Methyl decanoate	IS-6	4	24.117	74	87	5	0.000498-9.95	y=0.6335x+0.0037	0.985	66	1604	108
					143	8		y= 1.211x-0.0084	0.989	19	(1590[d])	
					43	13		y=1.275x-0.0054	0.995	29		
Ethyl decanoate	IS-6	5	25.007	88	101	5	0.022-441	y=0.2662x+0.0337	0.987	21	1649	245
					43	8		y=0.5430x-0.1734	0.995	13	(1641[a])	
						13		y=0.5400x-0.0071	0.991	17		
Isoamyl octanoate	IS-5	5	25.54	70	127	5	0.0001-2.23	y=2.4550x-0.0062	0.977	16	1676	267
					43	8		y=6.3390x-0.0213	0.968	13	(1689[a])	

Compound	IS	SIM group	Retention time (minutes)	Target ion (m/z)	Qualifier ion (m/z)%	Ethanol (% v/v)	Calibration range (µg/L)	Calibration Equation	r^2	SN ratio at LOQ	RI	Boiling Point (°C)[e]
						13		y=6.5900x-0.0166	0.963	22		
3-(methylthio)-1-propanol	IS-4	5	26.88	106	61	5	0.542-10857	y=0.0031x-0.0939	0.978	34	1745	90
					73	8		y=0.0068-0.1821	0.982	38	(1745[d])	
					31	13		y=0.0052-0.1022	0.975	57		
β-phenyl ethyl acetate	IS-1	6	28.75	104	91	5	0.004-858	y=369.5x-4.596	0.992	60	1847	229
					105	8		y=536.0x-8.695	0.993	44	(1803[c])	
					43	13		y=339.5x-4.576	0.995	86		
Ethyl dodecanoate	IS-6	6	29.01	88	101	5	0.001-20.0	y=0.2478x+0.0023	0.995	68	1862	269
					29	8		y=1.2620x-0.0269	0.988	42	(1849[a])	
					43	13		y=1.4360x-0.0215	0.982	46		
Geraniol	IS-7	6	29.13	69	93	5	0.016-331	y=4.674x-0.2193	0.989	38	1869	230
					68	8		y=4.558x-0.2447	0.994	21	(1862[d])	
					41	13		y=3.365x-0.0663	0.996	27		
β-phenyl ethanol	IS-7	7	30.485	91	122	5	8.36-167350	y=318.3x+17.32	0.98	87	1946	219
					65	8		y=286.4x+17.44	0.974	108	(1904[a])	
						13		y=324.9x+16.38	0.985	69		
Octanoic acid	IS-7	7	32.93	60	73	5	0.65-13016	y=1.5960x-0.1795	0.999	90	2090	240
					55	8		y=1.4110x+0.0526	0.993	40	(2096[a])	
					85	13		y=1.3080x-2.427	0.985	45		
Decanoic acid	IS-7	8	36.1	60	73	5	0.1-2301	y=4.042x-1.5080	0.981	25	2296	268
					57	8		y=3.352x-1.2580	0.993	36	(2370[a])	
						13		y=1.4950x-0.8231	0.985	53		
Vanillin	IS-7	9	41.9	151	109	5	0.03-696	y=0.0283x+0.0036	0.987	6.1	2621	285

Compound	IS	SIM group	Retention time (minutes)	Target ion (m/z)	Qualifier ion (m/z)%	Ethanol (% v/v)	Calibration range (μg/L)	Calibration Equation	r^2	SN ratio at LOQ	RI	Boiling Point (°C)[e]
					81	8		y=0.0226x+0.0018	0.994	3.6	(2555[c])	
					123	13		y=0.0368x+0.0003	0.995	4.5		
4-methyl-2-pentanone (IS-1)		1	9.43	43	58							
					85							
					106							
D5-ethyl butyrate (IS-2)		1	10.23	93	71							
					74							
					43							
D5-ethyl hexanoate (IS-3)		2	15.15	93	99							
					74							
					106							
2-Octanol (IS-4)		3	20.31	45	55							
					97							
					69							
D5-ethyl octanoate (IS-5)		3	20.82	93	106							
					74							
D5-ethyl decanaote (IS-6)		5	25.15	93	106							
					74							
					61							
D6-phenol (IS-7)		7	30.35	99	71							

[a] Rebière, L.; Clark, A.C.; Schmidtke, L.M.; Prenzler, P.D.; Scollary, G.R. A robust method for quantification of volatile compounds within and between vintages using headspace-solid-phase micro-extraction coupled with gc-ms - application on semillon wines. *Anal. Chim. Acta* **2010**, *660*, 149-157.

[b] Escudero, A.; Campo, E.; Fariña, L.; Cacho, J.; Ferreira, V. Analytical characterization of the aroma of five premium red wines. Insights into the role of odor families and the concept of fruitiness of wines. *J. Agric. Food. Chem.* **2007**, *55*, 4501-4510.

[c] Barata, A.; Campo, E.; Malfeito-Ferreira, M.; Loureiro, V.; Cacho, J.; Ferreira, V. Analytical and sensorial characterization of the aroma of wines produced with sour rotten grapes using gc-o and gc-ms: Identification of key aroma compounds. *J. Agric. Food. Chem.* **2011**, *59*, 2543-2553.

[d] El-Sayed, A.M. The pherobase: Database of insect pheromones and semiochemicals. http://www.pherobase.com/database/kovats/kovats-index.php (07/05/2010)

[e] Clarke, R.J.; Bakker, J. *Wine flavour chemistry*. Blackwell: Oxford, U.K., 2004.
